# Enhanced Photocatalytic Degradation of Methylene Blue by WO_3_ Nanoparticles Under NIR Light Irradiation

**DOI:** 10.3389/fchem.2021.683765

**Published:** 2021-07-01

**Authors:** Xiuzhao Yin, Lu Liu, Fujin Ai

**Affiliations:** ^1^ College of Health Science and Environmental Engineering, Shenzhen Technology University, Shenzhen, China; ^2^ Department of Civil and Environmental Engineering, Hanyang University, Seoul, South Korea

**Keywords:** photocatalysts, degradation, near-infrared radiation, photothermal effect, photocatalysts

## Abstract

Photocatalysts have been paid great attention owing to their excellent performance in the degradation of dangerous organic pollutants. Herein, a novel longitudinally grown WO_3_ photocatalyst was prepared by using a hydrothermal process, which had strong ultraviolet, visible light absorption, and weak near-infrared (NIR) absorption. The WO_3_ photocatalyst exhibited excellent performance in the rapid degradation of methylene blue (MB) in industry. The photothermal effect is mainly responsible for the rapid degradation of MB under NIR laser irradiation. Besides, different morphologies and structures affect the degradation of MB. The longitudinally grown enlarged the contact area between photocatalyst and MB, and expanded the scope of the absorption wavelength of light, enhancing the stability of photocatalytic materials. So this unique transverse longitudinal structure exhibited a potential capability for degrading organic pollutants.

## Introduction

Photocatalysts plays an important role in the field of environmental protection owing to their excellent performance, which have ability to oxidize organic and inorganic substrates in the dangerous organic pollutants (Fessi et al., 2020; He et al., 2020; Yu et al., 2021; Zhou et al., 2021). The unique structure has better property, which is used in the degradation of toxic chemicals and dyes by adsorption, biological degradation, chlorination, and ozonation from industrial wastewater (Gao et al., 2018). Among the nanostructured photocatalysts, the semiconductor is discrete, the valence band (VB) and the conduction band (CB) between a forbidden band, when energy is higher than the semiconductor absorption threshold of light, semiconductor material carrier separation, valence electrons interband transitions to produce photoelectron and holes (Ai et al., 2020) and then holes and electronics or molecules and ions, the form has the reducing or oxidizing activity of free radicals, the active free radicals to macromolecular organic matter degradation, carbon dioxide, water or another small molecule organic matter (Wang et al., 2016; Fessi et al., 2020). In the whole reaction process, photocatalyst itself does not have any change, the valence band holes show strong oxidation ability and the role of conducting electrons act as reductant (Hu et al., 2014).

In recent years, the study of semiconductor materials as photocatalysts for removing organic and inorganic substances in the aqueous phase has attracted extensive attention. Through absorbing photons with energy equal to or greater than the semiconductor bandgap, photocatalysis is carried out on the semiconductor photocatalyst to produce electron-hole (e^−^/h^+^) pairs, which extremely influnce quantum efficiency. The light absorption range of semiconductors is wide, which leads to high utilization efficiency of sunlight. However, the high recombination rate of photogenerated electrons and holes will reduce the photogenerated quantum efficiency. On the outer surface of the catalyst, the excited electrons and holes can undergo redox reactions with adsorbed substances (such as water, hydroxide ions (OH^−^), organic compounds, or oxygen). Generally the most important form of photocatalysis is free radicals. The charge can directly react with the adsorbed contaminants or water to generate free radicals, which in turn react with the contaminated substrate. But it’s much more likely to react with water, because there are more water molecules in sewage than contaminant molecules. The holes can oxidize water or OH^−^ to generate hydroxyl radicals (Ohbuchi, 2003; Umar and Aziz, 2013), which rapidly degrade the contaminants on the surface of the photocatalyst as well as contaminants in the solution. Another important electron conduction reaction is the reduction of adsorbed O_2_ to peroxide radicals. This reduces the recombination of electrons to holes and accumulates peroxy radicals, which are involved in degrading pollutants (Wei et al., 2021).

WO_3_ has the advantages of the narrow bandgap, low cost, non-toxic and stability under acidic and oxidation conditions. Due to wide range of band gap (2.4–3.5 eV), it can absorb up to 480 nm of visible lightand has a broad application prospect in the field of visible light catalysis (Lee et al., 2020). WO_3_ is generally a powdery solid with yellow color and is insoluble in water. The common morphologies include nanoparticles, nanowires, nanosheets and nanospheres. Different WO_3_ dimensions can be formed by different methods, which are 0 dimensional (0D) WO_3_, 1 dimensional (1D) WO_3_, 2 dimensional (2D) WO_3,_ and 3 dimensional (3D) WO_3_ respectively. Different dimensions have different characteristics. 0D WO_3_, monodisperse monoclinic WO_3_ quantum dots can be prepared by decomposition of ammonium tungstate oxide complex synthesized by hydrazine hydrate and WCl_6_ under hydrothermal conditions (Hailing and Sang-Bing, 2017). Through adjusting the reaction temperature, the particle size distribution of WO_3_-x QD_S_ can be precisely controlled within the range of 1.3–4.5 nm (Cao et al., 2015). One dimenbsional (1D) WO_3_ is common and easy to synthesize. Nowdays, different structure of (1D) WO_3_ was found, which include structure of nanofibers, nanotubes, nanorods, and nanowiresand two-dimensional (2D) WO_3,_ thin films, nanosheets, nanoplates etc (2D) WO_3_ have attracted extensive attention due to their high surface volume ratio, modulated surface activity, surface polarization, and oxygen-rich vacancies. Most 2D WO_3_ structures are thin films. Three-dimensional (3D) WO_3_ is usually a layered structure assembled from nanoparticles, nanoplates, nanorods, and nanosheets, usually showing as microspheres, micro flowers, sea urchin-like structures, mesoporous structures, and other irregular structures. 3D WO_3_ has the advantages of large specific surface area, high porosity and unique morphology.

The NIR region (700–1,400 nm) is considered as a biological window as living cells, and tissues have a low light scattering and absorption of NIR radiation. The advantage of NIR radiation is translated light energy into the localized heat named the photothermal effect, which is rapidly employed in hyperthermia treatment to kill cancer cells (Li et al., 2015; Li et al., 2016; Dong et al., 2019). However, the photothermal effect could play a crucial role in the enhanced degradation of pollution. It has been identified that graphen-based nanocomposites are strong photothermal effect agents under exposure to NIR radiation (Vijayaraghavan et al., 2014; Zhang et al., 2015; Ou et al., 2021).

In this work, a novel longitudinally grown photocatalyst, WO_3_, was prepared by a traditional method using a hydrothermal process, which had strong ultraviolet, visible light absorption and weak near-infrared radiation (NIR) absorption. Actually, the produced WO_3_ photocatalyst exhibited excellent performance in the rapid degradation of methylene blue (MB) in industry. It has been proved that the photothermal effect is mainly responsible for the rapid degradation of MB under NIR laser irradiation loaded visible light. In addition, it has been found that different morphologies and structures affect the degradation of MB. The longitudinally grown enlarged the contact area between photocatalyst and MB, and expanded the scope of the absorption wavelength of light, enhancing the stability of photocatalytic materials. Thus, this unique transverse longitudinal structure exhibited a potential capability for degrading organic pollutants in industry.

## Experiments and Reagents

### Materials and Reagents

Sodium hydroxide (NaOH), hydrochloric acid (HCl), and Ethylene glycol were purchased from Beijing Chemical Reagent Company. Sodium Tungstate Dihydrate (Na_2_WO_4_ 2H_2_O) was obtained from Sinopharm Chemical Reagent Co., Ltd. and used without any purification.

### Synthesis of WO_3_ Nanoparticles

1 mM Na_2_WO_4_ 2H_2_O was dissolved in 30 ml water until all solids were disappeared, then HCl was added to adjust the pH of the solution (pH = 1.5) under vigorous stirring for 30 min. The mixture was heated to 180°C for 24 h. Subsequently, the black WO_3_ nanocrystals were collected by centrifugation and washed three times with distilled water and ethanol. Finally, put it in a vacuum drying chamber for 6 h.

### Characterization

Powder X-ray diffraction (XRD) for structural characterization was performed on a D/max-2550PC X-ray diffractometer (Rigaku, Japan). Scanning electron microscopy (SEM) was conducted on a JEM-2100F electron microscope at an acceleration voltage of 200 kV (JEOL, Japan). The UV-vis diffuse reflectance and absorption spectra were obtained from Lambda 35 spectrophotometer (PerkinElmer) and U-3100 spectrophotometer (Hitachi), respectively. The X-ray photoelectron spectra (XPS) were taken on a VG ESCALAB MK II electron spectrometer using Mg Kα (1,200 eV) as the excitation source. Thermal images were recorded using a FLIR T420 thermal camera.

### The Degrade of MB Activity

The photocatalytic activity of the WO_3_ nanomaterials was evaluated by the degradation efficiency of MB under UV light (UVIR, 90 W), visible light (Philips, 40 and 90 W), and NIR laser irradiation (Armlaser Inc. United States, 2 W cm^−2^, 808 nm). In each experiment, 10 mg of the catalyst was suspended in 100 ml of the aqueous solution of MB (10 mg L^−1^), and the suspension was magnetically stirred in the dark for 30 min to establish adsorption/desorption equilibrium of MB molecules on the surface of the catalyst. Subsequently, the mixture was transferred to a double-walled photocatalytic reactor with a water circulation system to maintain the reaction mixture at room temperature. Then the suspension was exposed to UV, visible and NIR irradiation individually. At a given interval of time, 5 ml of the suspension was taken out and centrifuged, and the concentration of MB was analyzed by measuring the absorbance at 664 nm using a UV-vis spectrophotometer.

### The Photothermal Effect of Degradation

The photothermal effect of the samples was evaluated by using an 808 nm NIR diode laser system (Armlaser Inc. United States) with an output power of 1 W cm^−2^. In each experiment, 1 ml of an aqueous dispersion sample was transferred into a 1 cm × 1 cm × 4 cm cuvette and illuminated with a NIR laser. Then, during the experiment, the thermocouple of the suspension was immersed in the reaction mixture, and the temperature rise of the suspension under laser irradiation was measured with a digital thermometer.

## Results and Discussion

### Preparation and Characterization of the WO_3_

**FIGURE 1:**
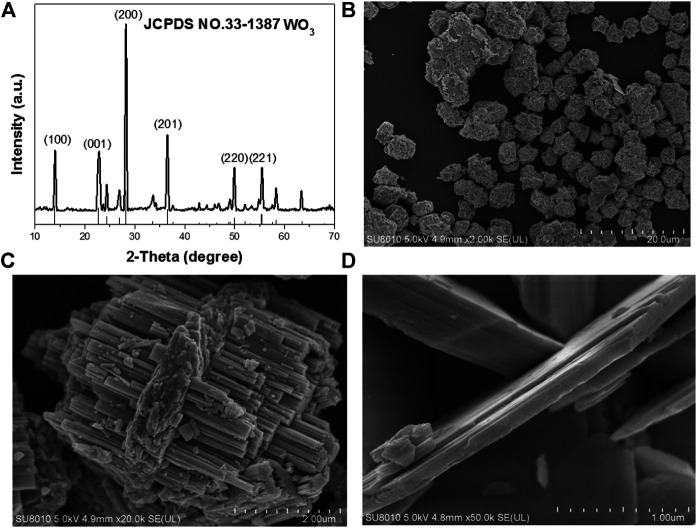
**(A)** Powder XRD patterns of the as-prepared nanocrystals and the standard WO_3_ powders. **(B)** The SEM images of the WO_3_ nanocrystals. **(C)** Typical SEM images of the WO_3_ nanocrystals. **(D)** Amplification SEM of WO_3_ nanocrystals.

The WO_3_ was synthesized by a hydrothermal method in the water environment. Firstly, Na_2_WO_4_ 2H_2_O was dissolved in water until all solids disappeared, then HCl was to adjust the pH of the solution under vigorous stirring. The mixture was heated to 180°C and maintained for 24 h. Subsequently, the black WO_3_ nanocrystals were collected by centrifugation and washed with distilled water and ethanol for three times. The single-crystalline nature of these NPs were confirmed by selected area electron diffraction (SAED) on individual dumbbells. The structure of as-synthesized NPs was further confirmed by X-ray diffraction (XRD) (Figure 1A). The pattern could be well indexed to the orthorhombic WO_3_ phase (JCPDS no. 33–1,387). The characteristic peaks of WO_3_ samples are consistent with those reported in the literature. The diffraction peaks at 2θ 13.9, 22.7, 28.1, 36.5, 49.9 and 55.5° correspond to the crystal planes of (100) (001) (200) (201) (220) and (221) respectively. The peak shape of WO_3_ is sharp, and there is no other diffraction peak, which indicate that the orthogonality crystal phase WO_3_ has high purity. The average edge length and width of intersection are 2 μm (Figure 1B). A similar experiment can be further demonstrated by the higher magnification scanning electron microscopy (SEM) image (Figure 1C). Figure 1D is an enlarged photo of the broken part of WO_3_, from which we can see each WO_3_ nanosphere in the empty microspheres. This novel longitudinal growth structure is very effective in photocatalysis.

### The Performance of Photocatalytic

**FIGURE 2:**
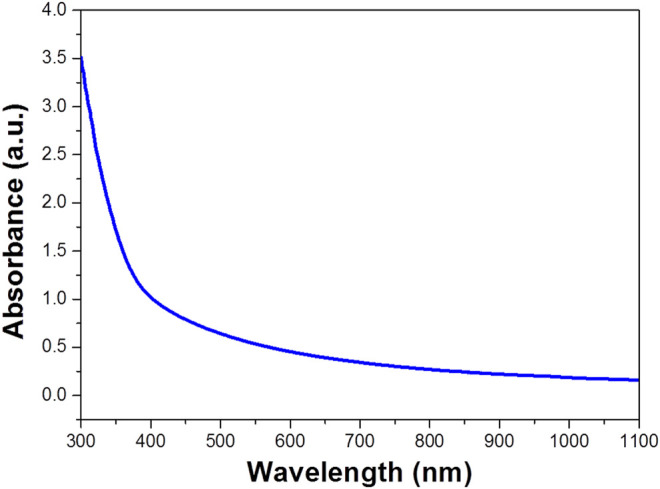
UV-vis-NIR absorption spectra of WO_3_ photocatalyst (concentration is 100 ppm).

Figure 2 shows the UV-vis-NIR absorption spectra of WO_3_ photocatalyst. The experimental data indicated that WO_3_ has great ultraviolet, visible light absorption and weak NIR absorption. It can be used as a photocatalyst which may be used for photocatalytic degradation of organic pollutions (Hui et al., 2019). We also predicted the photothermal effect under NIR laser and applied in enhanced degradation (Su et al., 2018) (Yao et al., 2016).

According to Lambert–Beer law

$${\text{A}=\text{l}}_{\text{g}}\left(\frac{{\text{I}}_{\text{0}}}{\text{I}}\right)={\text{l}}_{\text{g}}\left(\frac{1}{\text{T}}\right)=\text{kcd}$$ (1)

Among, A is the absorbance of the dye; I and I_0_ represent the intensity of transmitted light and incident light respectively; T is the transmittance (the ratio of the transmitted light intensity to incident light intensity); K is the proportion coefficient of absorbed light; C is the actual concentration of sample solution; D is the transmittance thickness (optical path) of the solution tank. According to the formula, there is a linear relationship between the absorbance of the solution and the concentration of the dye solution, which provides an operational method for us to measure the concentration of the solution by the absorbance. Therefore, standard solutions with different concentrations of dyes need to be configured before the experiment. After the absorbance is measured, the absorbance-concentration relationship curve of the standard solution is drawn and the linear relationship between absorbance and concentration is fitted according to the data. Using this linear relationship, the absorbance of the dye can be measured and then converted to the concentration. Then, the degradation rate calculation formula can be used to calculate the dye degradation efficiency D of the sample, as follows:

$$\text{D}=\frac{\left({\text{C}}_{\text{0}}-{\text{C}}_{\text{t}}\right)}{{\text{C}}_{\text{0}}}\times 100\%$$ (2)

**FIGURE 3:**
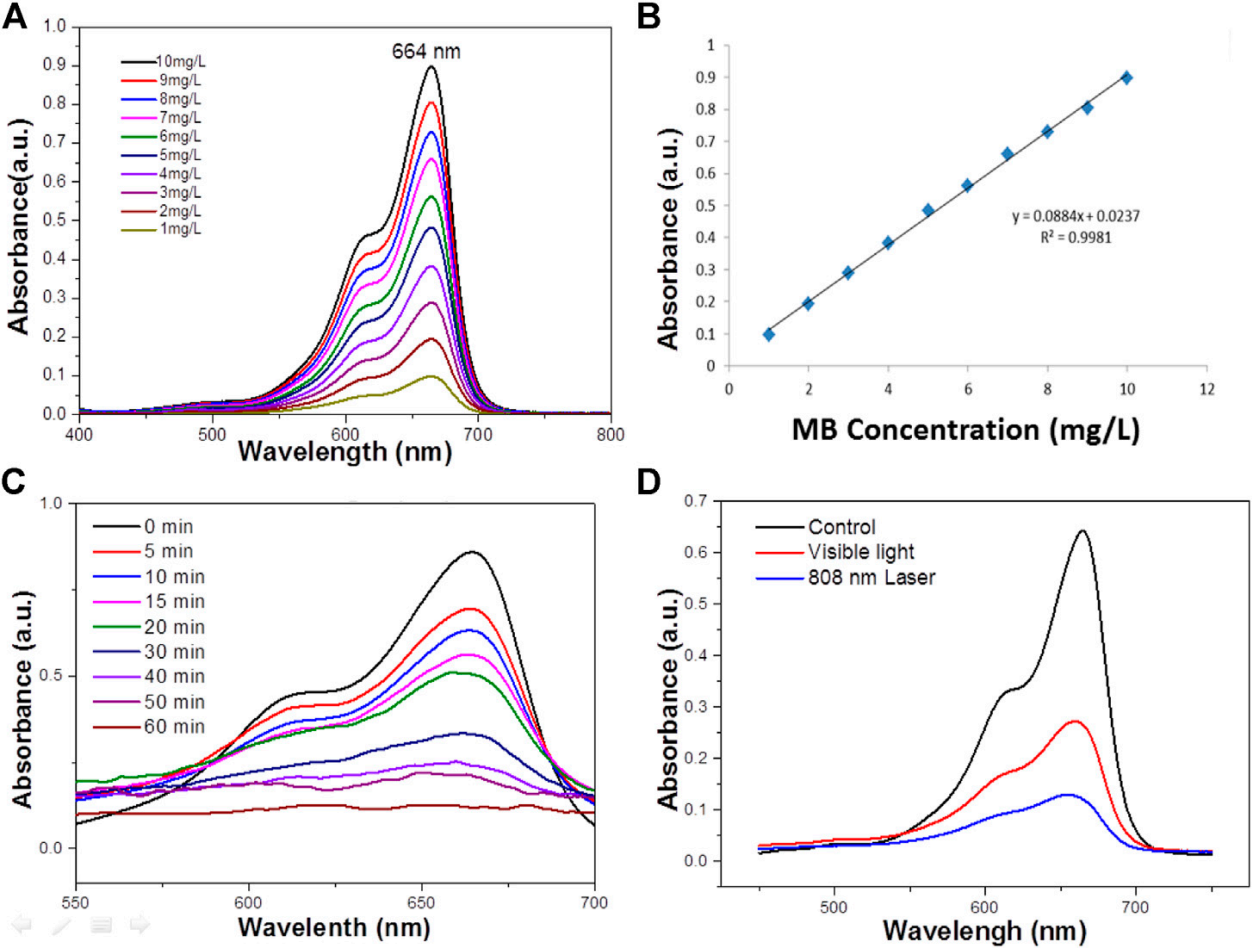
**(A)** The absorbance spectra of MB standard solutions of different concentrations. **(B)** The Absorbance-concentration fitting curve of MB solution. **(C)** UV–vis absorbance spectrum for the aqueous dispersion of the WO_3_ NPS under NIR light (concentration 100 ppm). **(D)** The photodegradation profile of MB in the presence of the WO_3_ nanocatalyst under exposure to visible light and 808 nm laser irradiation (concentration 100 ppm).

Where C_0_ is the concentration of the dye solution at adsorption equilibrium, and C_t_ is the concentration of the solution at the time of light t. At room temperature, a certain mass of MB is dissolved in deionized water in advance, and it is completely dissolved by ultrasonic for 3–5 min then the high concentration solution drainage transferred to the 1 L volumetric flask The washing solution is transferred to the volumetric flask with a glass rod in the beaker. The glass rod is cleaned three times, and the deionized water head is added into the scale line of the volumetric flask with a gutta percha, Rinse with solution and shake well solution. To improve the accuracy of the standard curve, 50 mg L^−1^ of MB solution was prepared as standard solutions according to the above methods to reduce the concentration error, and then diluted into 1–10 mg L^−1^ diluted solution to measured their absorbance. The absorbance spectrum curves of the standard solution are shown in Figure 3A. It can be seen that the characteristic absorption peaks of MB are located at 664 nm, which is due to the different chromogenic groups corresponding to different dyes, so the corresponding absorption wave positions of the characteristic peaks are different. The absorbance values corresponding to the characteristic absorption peak positions of the above dyes with different concentrations are shown in Figure 3B.

Water at high temperatures can promote a favorable condition for the cleavage of heterolytic bonds of the functional groups such as–OH and C=O persisting over the surface of WO_3_ NPs. Therefore, it is obvious that the characteristic band of MB located around 664 nm has completely disappeared, which suggests the structural rupture of MB molecules and their subsequent degradation by the action of the WO_3_ nanocatalyst. Moreover, the degradation of MB in the presence of NIR laser irradiation was compared with the degradation rate measured in visible (Figure 3D). It was found that MB was completely degraded at 100 min in the presence of visible light, which is significantly lower than the degradation rate found with the NIR laser. For comparison, the photo-degradation of MB was assessed under illumination by UV radiation also and it was observed that MB was entirely degraded in 80 min (Figure 3C). What’s more, the photodegradation of MB was very high in NIR radiation compared to both UV and visible radiation. The excellence of the WO_3_ NPS under exposure to a NIR laser could be related to the photothermal effect, which could be increased during the process of photocatalysis.

**FIGURE 4:**
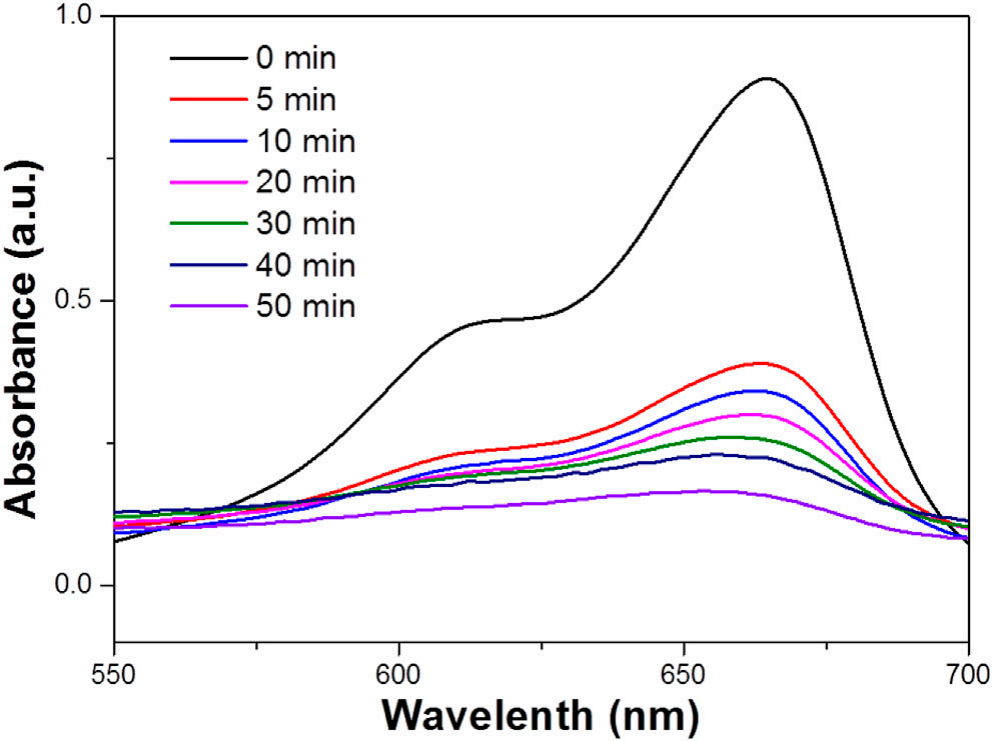
UV–vis absorbance spectrum for the aqueous dispersion of the WO_3_ NPs under UV light (concentration 100 ppm).

**FIGURE 5:**
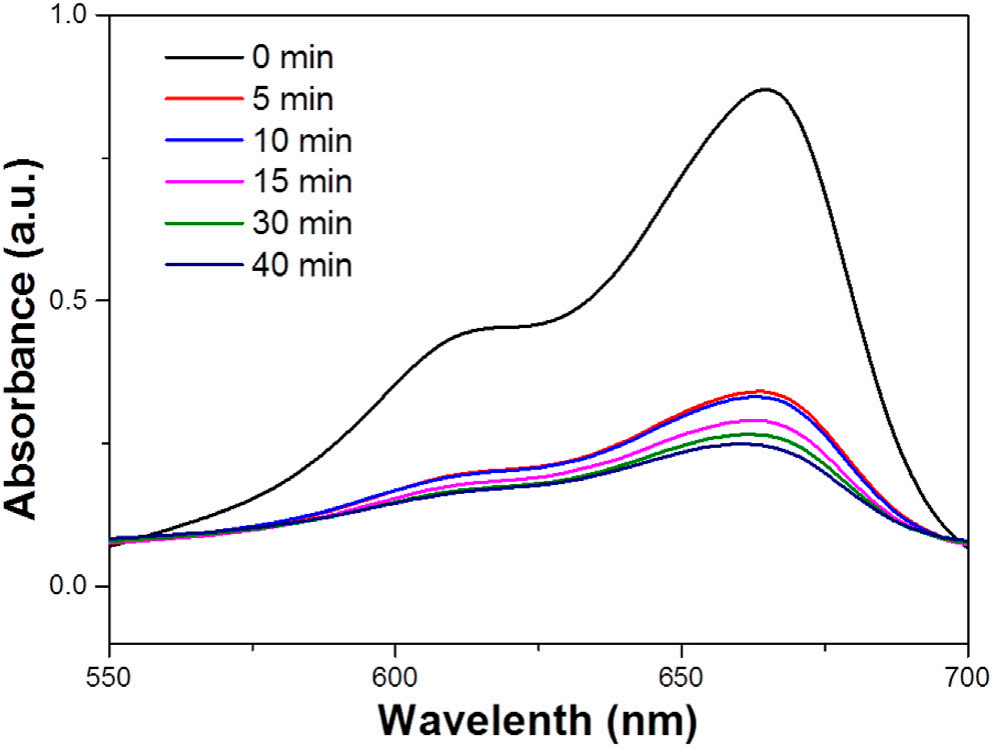
UV–vis absorbence spectrum for the aqueous dispersion of the WO_3_ NPs under visible light (concentration 100 ppm).

Furthermore, the degradation of MB in the presence of NIR laser irradiation was compared with the degradation rate under UV or visible laser irradiation. It was found that MO was degraded rate at 60 min in the presence of UV light, which is significantly lower than the degradation rate found with the NIR laser. Similar experiments have evaluated the degradation of MB under visible irradiation (Figure 4A), the degradation rate is lower than that under NIR laser. It was found that the influence of the WO_3_ photocatalyst under exposure to a NIR laser could be related to the photothermal effect, which could be generated during the process of photocatalysis. It is known that NIR radiation is mainly responsible for the photothermal effect. Therefore, to explore the influence of the photothermal effect which could play a decisive role in the endowed degradation of MB, this study has been further extended to emphasize the photothermal effect. It is identified that WO_3_ is a promising candidate for photothermal effect in the presence of NIR radiation (Ni et al., 2015). The degradation of MB was performed with a constant circulation of water, and even under this condition the photothermal effect could influence photodegradation, so we attempted to quantify the effect of the photothermal effect. Hot carrier relaxation that occurred in the WO_3_ NPs could be the reason for temperature rise under NIR laser irradiation (Neelgund and Oki, 2017). The relaxation of photo-generated carriers usually consists of several processes (i.e., carrier–carrier interaction, carrier–phonon scattering, Auger recombination, and carrier trapping) (Liu et al., 2013; Ji et al., 2016). It was reported that the carriers have a thermal distribution immediately after photo-excitation. Subsequently, carrier phonon scattering controls the energy relaxation and emits a large number of optical phonons, which makes the phonon distribution at the excitation point deviate greatly from the equilibrium distribution determined by the sample temperature. As the photothermal process is a collective electronic effect, the lattice temperature rapidly reaches equilibrium. Therefore, the hot carriers experience a much higher lattice temperature than the temperature of other parts of the sample, and the finite temperature variation does not influence the carrier dynamics (Diwald et al., 2004). This can lead to the photothermal effect and increase the local temperature of the photocatalytic mixture upon illumination by a NIR laser even under the condition of constant circulation of water. Hence compared with the degradation rate under UV and visible light, the photothermal effect led to the significant degradation of MB under NIR laser (Figure 5).

**FIGURE 6:**
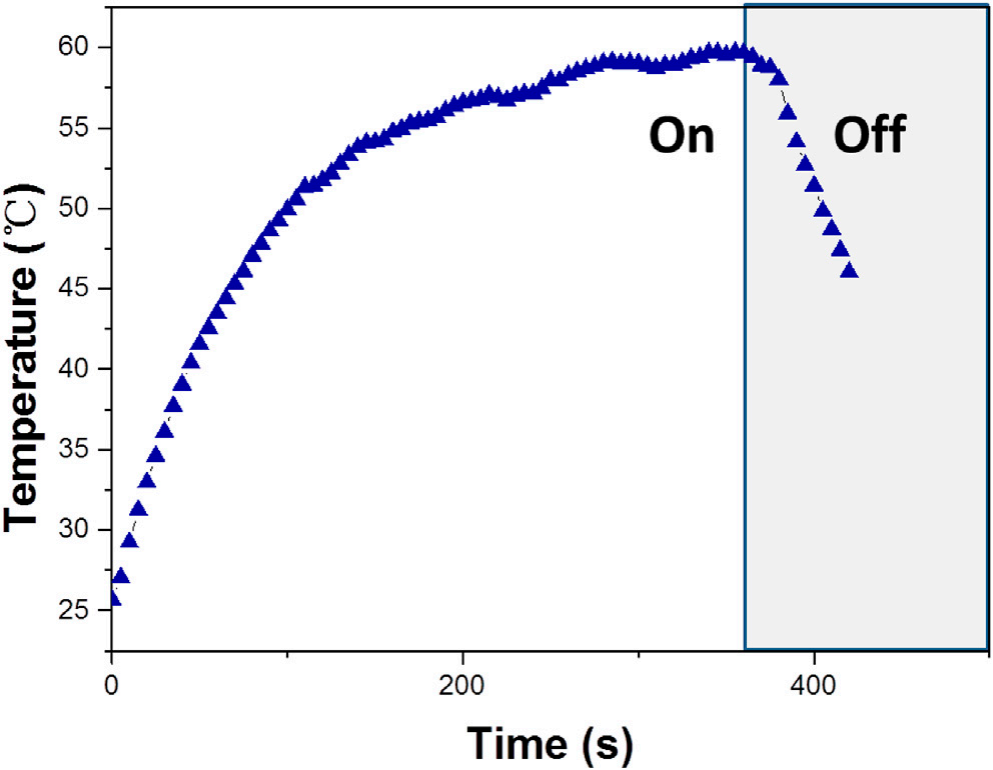
Temperature increase of WO_3_ measured under exposure to a 808 nm laser (concentration 100 ppm).

**FIGURE 7:**
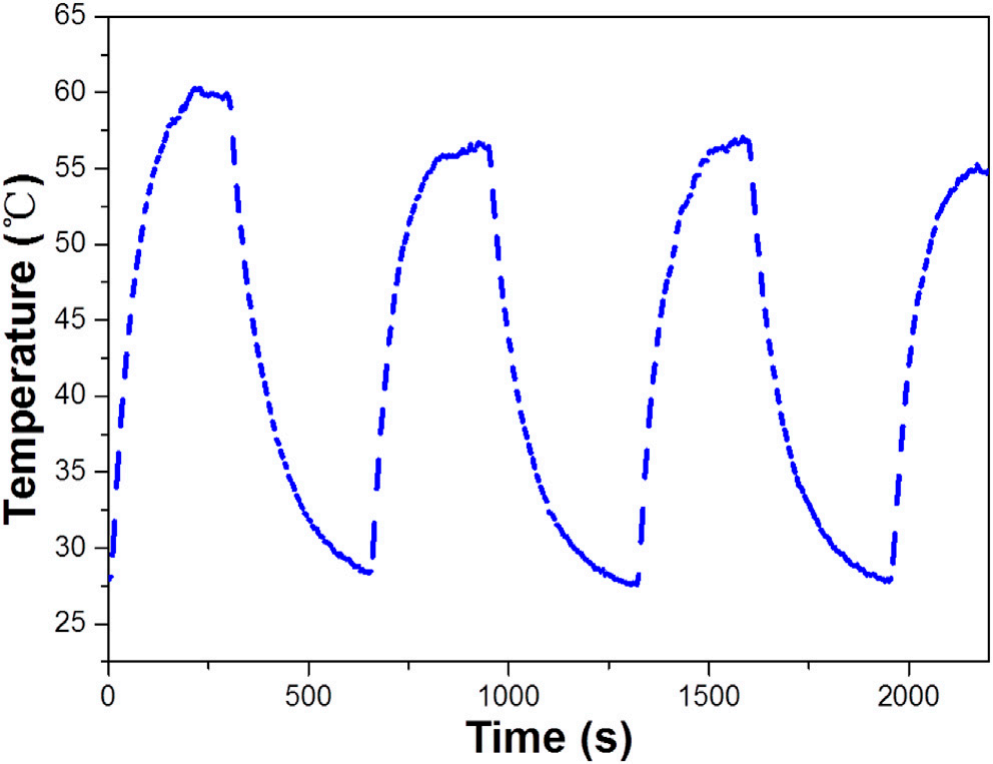
The temperature of WO_3_ measured for stability under exposure to a 808 nm laser (concentration 100 ppm).

Based on the importance of the photothermal effect in photocatalysis, the WO_3_ photocatalyst was quantified under exposure to 808 nm laser (Figure 6), illustrates the profile of time-dependent temperature increase in the samples in response to illumination of the 808 nm laser at their concentration level of 200 μg/ml. After 5 min of irradiation, an elevation of 33.4°C was found for an aqueous dispersion of the WO_3_ photocatalyst. In addition, the photothermal effect of the WO_3_ photocatalyst measured in five consecutive cycles was virtually constant, so no photobleaching was found in the WO_3_ photocatalyst (Figure 7).

The photothermal conversion efficiency of the WO_3_ photocatalyst was evaluated using the equation proposed by Roper with some modifications. For this purpose, the temperature change of the aqueous dispersion of WO_3_ photocatalyst (2 μg/ml) was measured as a function of time under exposure to the laser for 5 min, and at this juncture, the temperature rise reached a steady state. Then the laser was truned off to decrease the temperature, and the temperature was monitored to find heat transfer rate from the aqueous dispersion of the WO_3_ photocatalyst to the surrounding environment using eq:

$$\eta =\frac{\text{hS}\left(T_{\text{max}}-T_{\text{surr}}\right)-Q_{dis}}{I\left(1-10^{-A_{808}}\right)}$$ (3)

The Tmax (K) means the equilibrium temperature; Tsurr (K) is ambient temperature of the surroundings. The Qdis (W) is heat loss from light absorbed by the container, and it is calculated to be approximately equal to 0 mW. I (W·cm^−2^) represents incident laser power density; A808 is the absorbance of samples at 808 nm. Where h (W·cm^−2^·K^−1^) means heat transfer coefficient, S (cm^2^) represents the surface area of the container, the hS was calculated from Figure 2E. The hS is calculated using the following eq.

$$\tau_{s}=\frac{{\text{m}}_{\text{D}}c_{D}}{hs}$$ (4)

Hence, the photothermal conversion efficiency (Z) estimated for the WO_3_ photocatalyst was found to be 47.6%.

**FIGURE 8:**
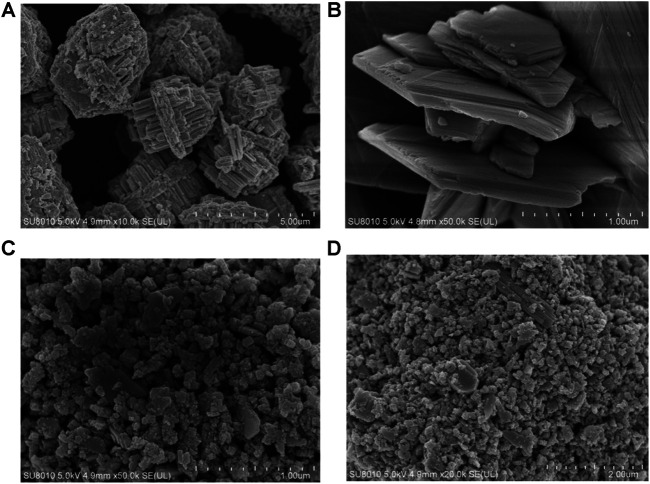
**(A,B)** Typical SEM images of the WO_3_ photocatalyst at 150°C. **(C,D)** Typical SEM images of the WO_3_ photocatalyst at 220°C.

Furthermore, the effects of different morphologies on MB degradation were also investigated. Figure 8 suggested that when the solvothermal temperature is 180°C and the concentration of Na_2_WO_4_.2H_2_O is 1 mg/ml, the morphology and particle size of the sample will be different when the solvothermal time is changed. When the time is 24 h, as shown in (Figure 8), the samples have uniform morphologies and even sizes. What’s more, the sample has a longitudinally grown photocatalyst, but there is a slight agglomeration phenomenon. When the temperature is 220°C, the sample particles were uniform and dense, and the agglomeration was serious. The reason may be that during solvothermal processes, the outer layer dissolves into tungsten material, which diffuses inward and is rich in voids, and then crystallizes on the inner particles. At the same time, the consumed shell is replenished by the crystallization of tungsten in the outer solution. In this repeated dissolution-diffusion-crystallization process, the tungsten element in the synthetic solution is transformed into the sphere and filled with voids.

**FIGURE 9:**
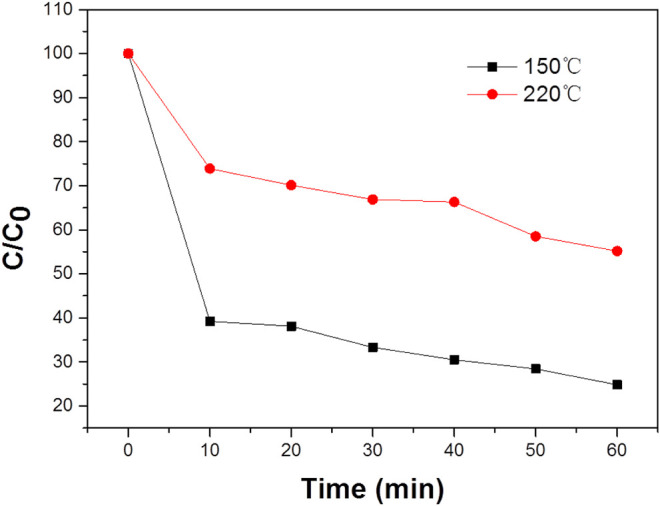
The photodegradation profile of MB in the presence of the WO_3_ photocatalyst under NIR laser irradiation.

When the raw material concentration is 1 mg/ml, the solvothermal temperature is 180°C, and the solvothermal time is 24 h, the morphology of the sample is the best, and the WO_3_ particles are the most uniform. The photodegradation performance of the sample is also the best. Figure 9 shows the photocatalytic degradation of MB solution under NIR light. This figure shows the photodegradation of MB solution after 30min dark adsorption by adding 0.329 g of WO_3_ photocatalytic material and simulating natural light irradiation. The typical absorption peak of MB is 664 nm. During the irradiation, the color of the MB solution decreased with increase of reaction time.

### The Mechanism of Enhanced Degradation

When WO_3_ is mixed with MB solution, a beam of light with an energy greater than the band gap of WO_3_ excites it, and the electrons in the valence band of WO_3_ jump to the conduction band, leaving a hole in the valence band. A hole has a strong oxidizability, which can be captured by the H_2_O ionization of hydroxyl ions to form hydroxyl free radicals (Li, 2004; Wu et al., 2020). It also has strong oxidizability, light raw electrons on the surface ofWO_3_ convert O_2_ to the superoxide free radicals, The light electrons and holes, hydroxyl radicals and superoxide free radicals react with the target pollutants by redox reaction, the target completely degraded into H_2_O and CO_2_. Another part of the electron-hole pairs can recombine themselves or with other photogenerated electrons or holes in the interior or surface, which will greatly reduce the photocatalytic efficiency of the photocatalyst (Hernández-Moreno et al., 2021; Wang et al., 2021).

**FIGURE 10:**
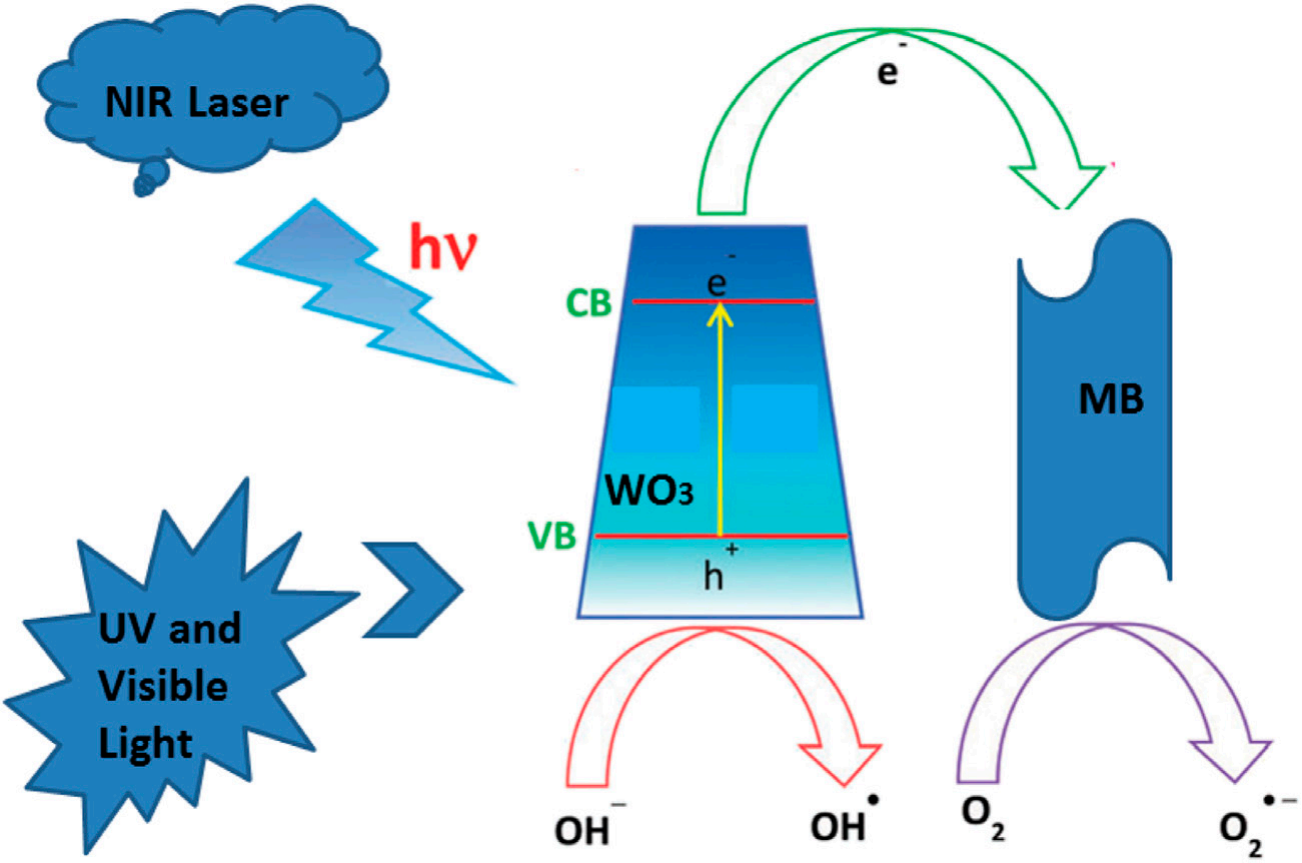
The mechanism for the degradation of MB in the presence of the WO_3_ NPs under NIR laser.

The degradation of MB employing the WO_3_ photocatalyst could be ascribed to its high surface area under NIR laser irradiation loaded UV and visible lightThe adsorption of MB molecules due to the p–p interaction between the aromatic ring of the MB molecules and WO_3_ photocatalyst enhances the adsorption of MB, which leads to the non-covalent adsorption of dye molecules (Loka and Lee, 2021). The existing research shown that the transition metal sulfides have unique physical and photoelectric properties, which can be used as a novel and efficient catalyst. Because the valence band generally consists of Sp3, it’s relative to the O2p orbital energy level is more negative, therefore, relative to the oxide of transition metal sulfides band gaps can be smaller and more likely to be sparked by the visible light, so it has potential application prospect in the field of photocatalytic oxidation. The photocatalytic activity mechanism of WO_3_ photocatalyst for MB degradation under near infrared laser irradiation is shown in Figure 10. The aqueous solution of WO_3_ photocatalyst and MB is exposed to light to produce electron pair (e) and hole (h +), and the photothermal effect promotes the electron from the valence band to the conduction band by leaving the hole in the valence band. This process remarkably reduces the combined probability of photo-excited electrons and holes in the WO_3_ photocatalyst. Moreover, a large number of photo-excited holes were retained to a large extent, which participate in the oxidation of MB and enhances photocatalytic activity. What’s more, more photo-generated holes can react with adsorbed water to form a hydroxyl radical (OH) (Mukhtar et al., 2021), which promotes the decomposition of MB. In addition, the oxygen adsorbed on the surface of MB can accept electrons and form superoxide radical anions (O_2_), which also results in the formation of OH upon protonation. These OH and O_2_ are responsible for the decomposition of MB under light irradiation (Zhang et al., 2010; Liu et al., 2013; Tang et al., 2020).

## Conclusion

In summary, WO_3_, a novel longitudinally grew photocatalyst, was prepared by a traditional hydrothermal method, which had strong ultraviolet, visible light absorption and weak near-infrared radiation absorption. The prepared WO_3_ photocatalyst has excellent degradation performance for MB in industry. It has been proved that the photothermal effect is mainly reason for the rapid degradation of MB under NIR laser visible irradiation. In addition, it was found that different morphologies and structures affect the degradation of MB. The longitudinally grown enlarged the contact area between photocatalyst and MB, and expanded the scope of the absorption wavelength of light, enhancing the stability of photocatalytic materials. Therefore, this unique transverse longitudinal structure exhibited a potential capability to degrade organic pollutants in industry.

## Data Availability

The original contributions presented in the study are included in the article/Supplementary Material, further inquiries can be directed to the corresponding author.
